# Mimicked Mixing-Induced Heterogeneities of Industrial Bioreactors Stimulate Long-Lasting Adaption Programs in Ethanol-Producing Yeasts

**DOI:** 10.3390/genes14050997

**Published:** 2023-04-27

**Authors:** Steven Minden, Maria Aniolek, Henk Noorman, Ralf Takors

**Affiliations:** 1 Institute of Biochemical Engineering, University of Stuttgart, 70569 Stuttgart, Germany; 2 Royal DSM, 2613 AX Delft, The Netherlands; 3 Department of Biotechnology, Delft University of Technology, 2628 CD Delft, The Netherlands

**Keywords:** scale-up, scale-down, bioreactor, stimulus–response experiment, substrate gradient, *Saccharomyces cerevisiae*, Ethanol Red™, transcriptomics

## Abstract

Commercial-scale bioreactors create an unnatural environment for microbes from an evolutionary point of view. Mixing insufficiencies expose individual cells to fluctuating nutrient concentrations on a second-to-minute scale while transcriptional and translational capacities limit the microbial adaptation time from minutes to hours. This mismatch carries the risk of inadequate adaptation effects, especially considering that nutrients are available at optimal concentrations on average. Consequently, industrial bioprocesses that strive to maintain microbes in a phenotypic sweet spot, during lab-scale development, might suffer performance losses when said adaptive misconfigurations arise during scale-up. Here, we investigated the influence of fluctuating glucose availability on the gene-expression profile in the industrial yeast Ethanol Red™. The stimulus–response experiment introduced 2 min glucose depletion phases to cells growing under glucose limitation in a chemostat. Even though Ethanol Red™ displayed robust growth and productivity, a single 2 min depletion of glucose transiently triggered the environmental stress response. Furthermore, a new growth phenotype with an increased ribosome portfolio emerged after complete adaptation to recurring glucose shortages. The results of this study serve a twofold purpose. First, it highlights the necessity to consider the large-scale environment already at the experimental development stage, even when process-related stressors are moderate. Second, it allowed the deduction of strain engineering guidelines to optimize the genetic background of large-scale production hosts.

## 1. Introduction

Microbial fitness is determined by the ability to maintain internal homeostasis in view of external heterogeneity. Complex sensory systems allow microorganisms to adapt to the resource availability in a given habitat for survival and enabling growth [1]. Stress-response mechanisms take over if environmental conditions turn for the worse. Depending on the severity of external stress, a growing organism might reduce proliferation, enter a quiescent state or even undergo self-induced cell death [2]. Nonetheless, the early response usually involves a transcriptional adjustment that represses growth capacities to save resources for adequate adaptation. This program is a conserved feature across species, commonly referred to as the stringent response or environmental stress response (ESR) in prokaryotes and eukaryotes, respectively [3,4]. Upon initiation, the transcriptional information propagates towards phenotypic change, which is well-aligned with the environmental shift.

In industrial fermentation development, in the lab the microbial habitat is that of a tightly regulated bioreactor. Several variables, such as pH, temperature, dissolved oxygen and substrate concentration, are kept at optimal levels to maintain the microbial host in the physiological state of optimal productivity. Still, many bioprocesses suffer unforeseen performance losses when engineers transfer a process from the homogeneous lab environment to the industrial scale [5]. So-called biological scale-up effects occur when transport limitations in large tanks prevent proper mixing, cooling and mass transfer needs of the broth rendering the environment heterogeneous [6,7]. Often, limiting substrate concentrations are set during production phases to ensure that microbial activities still cope with the technical limits of aeration, heat exchange, etc. Such limiting substrate supply defines substrate-to-product conversion yields, and cellular and volumetric productivities. Gradients of said substrates evolve as their reaction time is typically shorter than the mean circulation time in industrially-sized tanks [8]. Therefore, a fluctuating physicochemical environment clashes with a complex biological sensory system. Understanding the systematic incompatibility helps to understand why biological scale-up effects occur and guide rational strain engineering efforts [9].

*Saccharomyces cerevisiae*, a widely adopted host in the biotech industry, is equipped with the sensory abilities to adapt to the entire spectrum of substrate concentrations it may encounter in a fermentation process. Backed by large-scale process data, simulation studies confirmed the existence of glucose concentration gradients spanning several metabolic regimes in a glucose-limited fed-batch production of baker’s yeast [10,11,12]. For instance, highly concentrated feed solutions may locally introduce glucose concentrations above respiratory capacities, potentially triggering carbon catabolite repression. Distant from the feed inlet, in turn, the substrate becomes depleted triggering starvation-like signals. However, minute-to-hour adaptation times typically exceed the second-to-minute exposure times in the stirred bioreactor space [13]. Thus, cells are prompted to initiate adaptive or even stress-responsive programs, and either their execution or trimming causes unnecessary resource expenditure that might even lead to phenotypic heterogeneity [13,14].

Following the scale-down route, researchers aim to use insights from physical large-scale studies to investigate the physiological response against realistic gradients. Especially in high-cell density processes, the influence of carbon starvation zones draws more and more attention [15,16,17,18]. Dedicated experiments with prokaryotic hosts revealed redundant induction and repression of the stringent response when cells were repeatedly withheld from the limiting substrate [19,20]. Derived knowledge on the gene-regulation level ultimately guided rational strain engineering approaches to increase microbial robustness [21,22]. In a recent study, we investigated the transcriptional profile of respiring *S. cerevisiae* against short-term transitions between glucose limitation and starvation in an analogous approach [23]. First-time exposure to acute glucose depletion elicited the ESR prematurely in a non-adapted culture, while it was globally repressed in a ‘stand-by mode’ enabling dynamic response once the population was adapted to the signals. We concluded that regulatory elements of the ESR, such as the involved transcriptional activators Msn2/4, might be promising targets for strain engineering approaches. However, both the culture conditions and the applied haploid CEN.PK 113-7D strain has little to no relevance in industrial fermentation processes. In addition, this strain harbors several non-synonymous mutations in its cAMP signaling system, the primary mediator of ESR activity [24]. Consequently, we set out to replicate the experiment with the diploid industrial Ethanol Red™ strain under anaerobic, ethanol-producing conditions.

## 2. Materials and Methods

### 2.1. Strain, Maintenance and Seed Culture

The commercial, MATa/MATα diploid *S. cerevisiae* strain Ethanol Red™, currently marketed by Fermentis (Lesaffre, Marcq-en-Barśul, France), was kindly provided by Royal DSM N.V. (Delft, The Netherlands). Cells were preserved in 30% (*v*/*v*) glycerol at −70 °C and grown on yeast extract peptone dextrose (YPD) agar plates for two days before starting the aerobic seed cultures. First, a 10 mL glass vial with 5 mL of YPD broth was inoculated with a single colony and incubated at +30 °C on an orbital shaker operated at 120 rpm for 6–8 h. Subsequently, the whole volume was pelleted and used to inoculate 110 mL of a synthetic medium in a 1000 mL baffled shake flask and grown under identical conditions overnight until the stationary phase was reached. The medium was designed to support approximately 5.0 g·L^−1^ biomass during carbon-limited growth with 50 g·L^−1^ glucose and contained 10 g·L^−1^ ammonium sulfate, 6.0 g·L^−1^ monopotassium phosphate, 1.0 mg·L^−1^ magnesium sulfate heptahydrate, 19.1 mg·L^−1^ ethylenediaminetetraacetic, 4.5 mg·L^−1^ zinc sulfate heptahydrate, 1.0 mg·L^−1^ manganese(II) chloride tetrahydrate, 0.3 mg·L^−1^ cobalt(II) chloride hexahydrate, 0.3 mg·L^−1^ copper(II) sulfate pentahydrate, 0.4 mg·L^−1^ sodium molybdate dihydrate, 4.5 mg·L^−1^ calcium chloride, 3.0 mg·L^−1^ iron(II) sulfate heptahydrate, 1.0 mg·L^−1^ boric acid, 0.1 mg·L^−1^ potassium iodide, 0.05 mg·L^−1^ D-biotin, 1.0 mg·L^−1^ calcium pantothenate, 1.0 mg·L^−1^ nicotinic acid, 25.0 mg·L^−1^ myo-inositol, 1.0 mg·L^−1^ thiamine HCl, 1.0 mg·L^−1^ pyridoxine HCl, 0.2 mg·L^−1^ para-aminobenzoic acid, 0.42 g·L^−1^ tween 80, 10 mg·L^−1^ ergosterol and 0.2 g·L^−1^ Struktol J 674 antifoam (Schill und Seilacher, Hamburg, Germany). The same medium was used for seed, batch and continuous cultures.

### 2.2. Chemostat Setup

Anaerobic cultivation experiments were carried out in a stainless-steel benchtop bioreactor (Bioengineering, Wald, Switzerland) with a liquid working volume of 1.7 l under a 0.3 bar overpressure. The reactor system and its rapid sampling device were operated as previously described [15,23] with the following modifications: (i) silicone tubing was replaced by oxygen-impermeable tubing (Norprene, Cole Parmer, Vernon Hills, IL, USA), (ii) anaerobiosis was maintained with a sterile nitrogen supply of 0.425 vvm and (iii) no antifoam agent was supplied as it was already present in the medium. Furthermore, the headspace of the feed casket was kept flushed with sterile nitrogen throughout the experiment.

The reactor was aseptically inoculated with 100 mL of seed culture and operated in batch mode until a decrease in CO_2_ emission indicated glucose exhaustion. Subsequently, the chemostat was initiated via continuous medium influx and broth efflux at net rates of 2.83 mL∙min^−1^ to yield a dilution rate (*D*) of 0.10 h^−1^. During stimulus–response experiments (SREs), the system was operated as an intermittently fed chemostat. The feeding pump was set to 0.00 mL∙min^−1^ for two minutes while the harvest pump control was inactive. In the case of repeated perturbation cycles (2 min feed off, 7 min feed on), the feed rate was set to 3.64 mL∙min^−1^ to maintain the same net *D*.

### 2.3. Stimulus–Response Design

**Figure 1:**
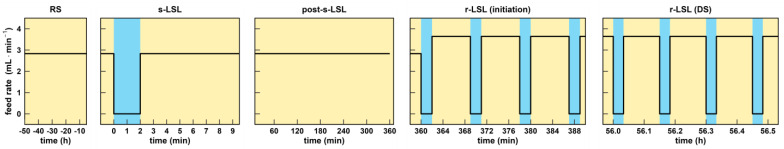
Process design of the chemostat experiment. DS, dynamic steady state; RS, reference steady state; r-LSL, repeated limitation–starvation–limitation transition; s-LSL, single limitation–starvation–limitation transition.

Three biologically independent fermentation experiments were carried out according to the process design depicted in Figure 1. Each chemostat operated for 5 residence times (*τ*) of constant *q*_carbon dioxide_ to sample the reference steady state (RS). Thereafter, a single limitation–starvation–limitation (s-LSL) stimulus was imposed to track the non-adapted response as a time series of up to six hours. Subsequently, the mode of operation changed to an intermittent feeding regime. After five *τ* of repeated cycling, the new, dynamic steady state (DS) was established. The adapted response was sampled as a time series during repetitive cycles (r-LSL) and thus limited to one representative nine-minute series. Dynamic steady state values were expressed as cycle averages.

### 2.4. Analytical Procedures

Sample processing and analysis are thoroughly reported in [15,23]. In brief, biomass, expressed as dry matter of biomass (*DMB*), was determined gravimetrically. All extracellular metabolites were determined with UV-based enzymatic kits (r-biopharm AG, Darmstadt, Germany). Intracellular carbohydrate and RNA pools were assessed according to the original protocols from Parrou and Sasano, respectively [25,26]. Unknown carbon in the supernatant was determined by subtracting the molar carbon concentrations of the antifoam agent and all quantified extracellular metabolites except CO_2_ from the total organic carbon concentration in the broth supernatant. We assumed no uptake of the antifoam agent, which has a carbon mass fraction of 61% (*w*/*w*) [27]. Total organic carbon was measured indirectly with a multi-N/C 2100 S composition analyzer (Analytik Jena, Jena, Germany) by reducing the inorganic carbon fraction from the total carbon fraction of the supernatant. We estimated a 4.8% loss of ethanol due to stripping which was accounted for in the carbon balance and parameter calculation (*Y*_ethanol/glucose_, and *q*_ethanol_). Ethanol stripping was estimated based on the approach by Löser and colleagues [28] and is described in detail in Supporting Information A1.

### 2.5. Processing of Next-Generation Sequencing Samples

We used the Quick-RNA Fungal/Bacterial Miniprep Kit (R2014, Zymo Research, Freiburg, Germany) for total RNA extraction with the following changes to the manufacturer’s instructions: 0.5 mL of the biosuspension was sampled directly into a ZR BashingBead™ lysis tubes, pre-loaded with 0.5 mL of a lysis buffer. After the sample was withdrawn, the whole tube was instantly flash-frozen in liquid nitrogen and stored at −70 °C. The extraction protocol was resumed by thawing the samples halfway (5–10 min at room temperature) before performing the homogenization step in a Precellys 24 tissue homogenizer (Bertin Technologies, Montigny-le-Bretonneux, France) twice for 20 s at maximum speed with a 10 s break in between. At the end of the protocol, total RNA was eluted with 60 μL DNase/RNase-free H_2_O and stored at −70 °C.

One 30 μL aliquot from each sample was shipped for mRNA sequencing to AZENTA/GENEWIZ (Leipzig, Germany). The contractor performed an initial quality check using Agilent 2100 BioAnalyzer (Agilent, Santa Clara, CA, USA) which revealed a heterogeneous RIN (RNA integrity number) value distribution ranging from 2.2–9.9 for all samples. After personal communication with the contractor, it was decided that the project would be commenced since the heterogeneous RIN values were a result of non-uniform rRNA peaks, even though the cause for this effect was unknown. Peaks for nucleotides of <1500 nt including mRNA, however, showed uniform distribution. Next, polyA-selected cDNA libraries were synthesized and sequenced as 150 bp paired-end reads on a NovaSeq 6000 platform (Illumina, CA, USA) with a sequencing depth of 2 × 10^7^ reads.

### 2.6. Gene Expression Analysis

A sequencing output in the form of *.fastqsanger* files was uploaded on a local Galaxy platform [29] followed by a quality check using *FastQC* v. 0.72 [30]. Sequence files were subsequently aligned with *TopHat* v. 2.1.1 [31] against the phylogenetically closely related *S. cerevisiae* S288C reference genome [32] (GCA 000146045.2-2011), which was accessed from the ENSEMBL database [33]. The overall alignment rate ranged between 83 and 92%. Genes were annotated to *S. cerevisiae.R64-1-1.50.gtf* (from ENSEMBL) and counted using *featureCounts* v. 1.6.4 [34]. From here, count tables were extracted from the Galaxy platform and merged into a *data.frame* object for further processing in the R environment v. 1.4.1106 (R Core Team 2021).

Differentially expressed genes (DEGs) were computed using *DESeq2* v. 1.32.0 [35], applying the likelihood-ratio test with threshold values for |log_2_-fold change| and a false discovery rate (FDR) [36] of 1.0 and 1 × 10^−3^, respectively. More detail is provided in Supporting Information A3 and the experimental design matrix is reported in Supporting Information B (sheet 1). Time series data was clustered with the *kmeans* function from the *stats* (v. 4.1.0) package and functional annotations were derived from the web implementation of *YeastEnrichr* [37,38]. The raw enrichment analysis output can be accessed in Supporting Information B. Gene set enrichment analysis (GSEA) was carried out using *GAGE* (v. 2.42.0) [39] with log_2_-scaled count tables (Supporting Information B, sheet 19) and pre-defined literature data sets (sheet 20) or transcription factor (TF) target sets (sheet 21), which were downloaded from the *Yeastract* database [40]. The results in Figure 6 were reduced to sets showing statistical significance (FDR < 1 × 10^−3^) during at least one condition in the SRE. Multiple set intersections of DEG lists were computed using the package *SuperExactTest* (v 1.1.0) which uses the combinatorial theory to provide the statistical significance of intersections [41].

## 3. Results

### 3.1. Characterization of Extracellular Glucose Profile

**Figure 2:**
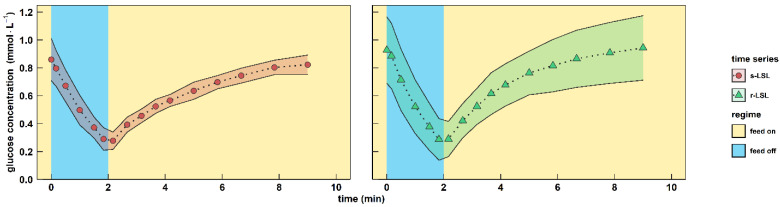
Characterization of the famine stimulus. Extracellular glucose concentrations during the course of one perturbation cycle are shown. Red circles indicate dynamics following a single (s) LSL transition (“feed off” phase) and green triangles indicate trends over a representative repetitive (r) LSL cycle during the DS. Time point 0 min of the s-LSL response is the equivalent of the RS. All values indicate means ± standard deviation of three biological replicates.

The showcasing stimulus–response experiment enabled the observation of transcriptional feedback mechanisms of the industrial yeast Ethanol Red™ (ScER) after intermittent carbon supply. After anaerobically growing cells were adapted to strict glucose limitation for five residence times in a chemostat, the glucose feed was stopped for two minutes to establish a single limitation–starvation–limitation (s-LSL) cycle. A sharp, uptake-driven drop of the glucose concentration from 0.86 mmol·L^−1^ to 0.28 mmol·L^−1^ occurred, which restored to previous steady-state levels within eight minutes after feed resumption (Figure 2, left panel). The biomass-specific glucose uptake rate (*q*_glucose_) ramped down from 45% to 21% of the maximum capacities (for *q*_glucose,max_, see Table 1). Glucose uptake kinetics remained also for cells that were completely adapted to repeated LSL cycling for five residence times (r-LSL, Figure 2, right panel). Notably, the perturbation never challenged cellular maintenance demands since the minimum *q*_glucose_ of 2.5 mmol·g*_DMB_*^−1^·h^−1^ stayed 5-fold above the maintenance rate of 0.5 mmol_glucose_·g*_DMB_*^−1^·h^−1^ [42].

### 3.2. The Physiology of Dynamic and Steady-State Adaptation toward Short-Lived Famine Stimuli

**Figure 3:**
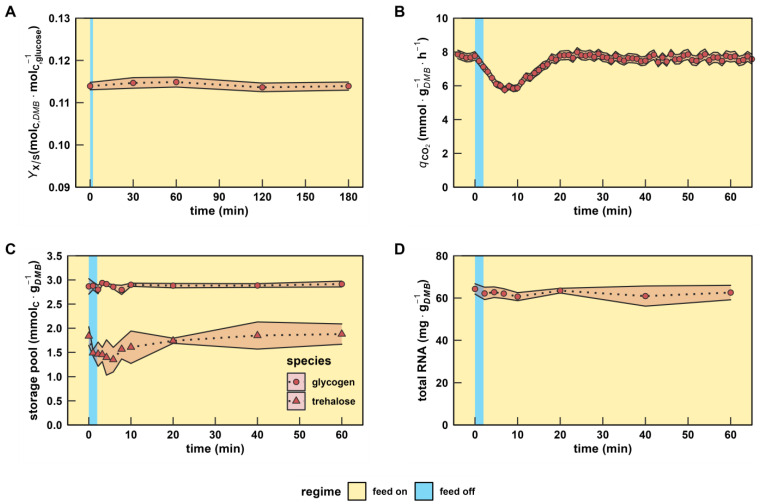
Characterization of macroscopic readouts after a single LSL stimulus. (**A**) Biomass-substrate yield up to 180 min. (**B**) Biomass-specific carbon dioxide production rate, (**C**) intracellular carbon storage, and (**D**) total RNA pool dynamics up to 60 min. The time series indicates dynamics following a single transition into the starvation phase (“feed off” phase). Time point 0 min is equal to the reference steady state. All values indicate means ± standard deviation of three biological replicates.

The biomass-substrate yield remained for three hours after the s-LSL cycle indicating the absence of growth-arresting measures by the non-adapted yeast culture (Figure 3A). Steadiness of growth was backed by constant intracellular RNA levels (Figure 3D, *p*-Value > 0.05) that may also serve as a surrogate parameter for ribosomal content [43]. Regarding primary metabolism, substrate shortage was propagated through glycolysis causing a transitory reduction of carbon dioxide emission from 7.8 ± 0.2 mmol·g*_DMB_*^−1^·h^−1^ to 5.8 ± 0.2 mmol·g*_DMB_*^−1^·h^−1^ (Figure 3B). We reason that the inertness of the off-gas measurement caused the five-minute delay between both minima of glucose uptake and CO_2_ emission. A similar observation was reported in a previous study with the same bioreactor system [15]. In addition, an acutely decreased glycolytic flux along the s-LSL trajectory caused the short-term mobilization of trehalose (*p*-Value 0.06–0.11), but not glycogen, during the first six minutes.

After the s-LSL cycle, repeated (r) r-LSL stimuli were performed during the second phase of the experiment. A single r-LSL cycle was analyzed using averaged data, the so-called dynamic steady state (DS). Therewith, distinct adaptations of ScER resource management were unraveled that mimicked cellular efforts to cope with the fluctuating substrate environment (Table 1). Supported by closing carbon recoveries, the biomass-substrate yield (*Y_DMB_*_/glucose_) dropped by 6.9% whereas the net dilution rate and glucose feed remained. Consequentially, an equal rise of biomass-specific glucose uptake occurred. Relative to the reference steady state (RS), the surplus of the glycolytic input was channeled towards CO_2_ emission. From the trend, increased ethanol production and glycerol secretion were also found, which agrees with stoichiometric expectations. The DS population released 71.2% more unknown carbon products than the reference state. Even though this value possesses low statistical confidence, the trend supports the slightly reduced *Y_DMB_*_/glucose_ and might point to elevated cell lysis [44]. Intracellular resource allocation changes made up the most pronounced r-LSL adaptations. We observed glycogen and trehalose pool size reductions of 42% and 32%, respectively. They were accompanied by increased intracellular RNA concentrations from 64.3 ± 2.5 mg·g*_DMB_*^−1^ to 80.7 ± 3.1 mg·g*_DMB_*^−1^. Even though it is a well-known tendency of *S. cerevisiae* to counterbalance ribosome abundance with the degradation of glycogen reserves, the correlation is anticipated to be growth-rate-dependent only [45].

In consequence, we set out to investigate whether or not the sensing of the dynamic extracellular environment triggered cascading effects that propagated through the ScER regulatory network in a manner that was independent on the growth rate.

### 3.3. The Transcriptional Response to Single Starvation Exposure (s-LSL)

**Figure 4:**
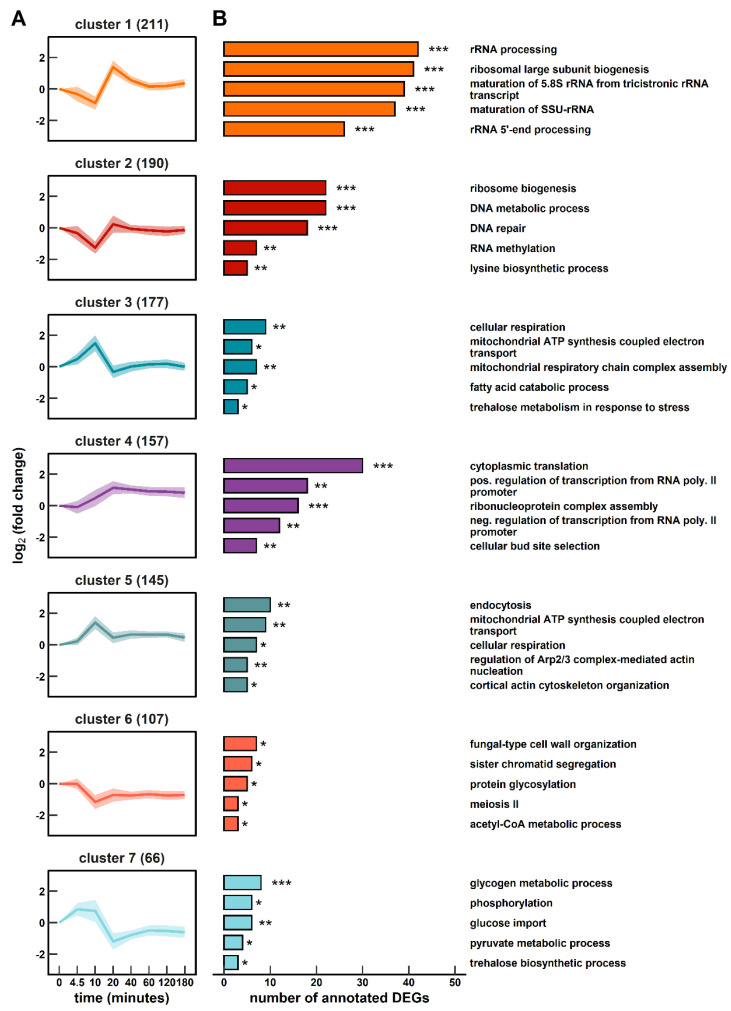
Gene expression dynamics following the single-LSL stimulus up to 180 min. (**A**) Seven k-means clustered groups of co-expressed genes are shown with the number of corresponding genes in brackets. (**B**) Corresponding gene ontology (GO) enrichment analysis. The false discovery rate (FDR) is indicated by asterisks for each GO term (* 1 × 10^−5^ ≤ FDR < 5 × 10^−2^; ** 1 × 10^−10^ ≤ FDR < 1 × 10^−5^; *** FDR < 1 × 10^−10^).

The post-s-LSL cycle monitoring of ScER cells that operated at the steady state with an industrially representative production rate [46] revealed a differential expression of 1053 genes (Figure 4). Co-regulated mRNAs were grouped into seven clusters containing 66 to 211 genes before characterizing them through functional enrichment. This non-adapted feedback peaked between 10–20 min and entirely relaxed 60 min after the stimulus added.

Clusters 1 and 2 followed a similar repression/de-repression trajectory with a strong amplitude of cluster 1 before RS levels were restored. Both clusters were significantly enriched with genes of the ribosome biogenesis (RiBi) ontology, which were further specified as sub-ontologies such as rRNA processing or subunit maturation. Notably, the observed expression changes did not result in a detectable correlation with total intracellular RNA levels (Figure 3D). Cytoplasmic translation was also enriched in the steadily induced cluster 4 opposing the downregulation trend of clusters 1 and 2 during early responses. Cluster 4 contains 29 of 37 differentially expressed ribosome subunits. Thus, the s-LSL response elicited 27% of all 135 ribosome proteins (RPs). However, the remaining majority responded only in a dampened manner according to the analysis of RiBi-associated gene expressions.

In addition to the well-equilibrated response of protein formation, we observed evidence of the transiently reduced production of cell cycle-related transcripts. Cluster 2 covers DNA metabolic and repair mechanisms while cluster 6 comprises sister chromatid segregation and the term “meiosis II”. Even though industrial diploid strains such as ScER should exhibit high mitotic stability [47], meiotic events especially during nutrient starvation are not uncommon [48]. In addition, the three genes leading to the significant call of “meiosis II”, namely *IRC15*, *IML3* and *IPL1*, are involved in both mitotic and meiotic processes [49].

Clusters 3 and 5 somewhat mirror the trends of clusters 1 and 2 in an opposite manner. As they comprise a significant proportion of genes encoding respiratory capacities, this is unexpected given the strictly anaerobic environment. However, factoring in other functional enrichments, the picture of an acutely energy scavenging population evolves. Upregulated mRNAs coding for both endocytic functions and related regulatory elements such as Arp2/3-mediated actin nucleation are well-studied responses of acute glucose withdrawal [50]. Furthermore, the joint analysis of clusters 3 and 7 reveals the induced metabolic activity of the major carbon storage compounds glycogen, trehalose, and fatty acids. Rapid trehalose mobilization was accompanied by a 1.2-fold induction of the neutral trehalase encoding transcript *NTH1* in agreement with the literature [51]. Conversely, glycogen mRNAs in cluster 7 were mainly involved in glycogen buildup (*GAC1*, *GIP2*, *GLC3*, *GLG1*, *GSY1*, *GSY2* and *UGP1*) whereas the respective polymer level remained constant. A functional dependency on the strategic upregulation of the respiratory apparatus becomes evident in genes that make up the “fatty acid catabolic process” ontology. In fact, products of transcripts such as *FOX2*, *ECI1*, *POT1* and *IDP3* catalyze the O_2_-dependent β-oxidation of fatty acids [52]. Glucose import was equally tuned by inducing three high-affinity facilitators (Hxt4/17/13) and two of three hexokinases (Hxk1 and Glk1) [53].

In essence, the cellular transcriptional program prepared the population for carbon scarcity. The reset occurred after the glucose availability improved again. Interestingly, even though the transcriptome was deemed to be fully relaxed after 60 min, clusters 4, 6, and 7 did not re-install RS levels.

### 3.4. The Transcriptional Response to r-LSL

**Figure 5:**
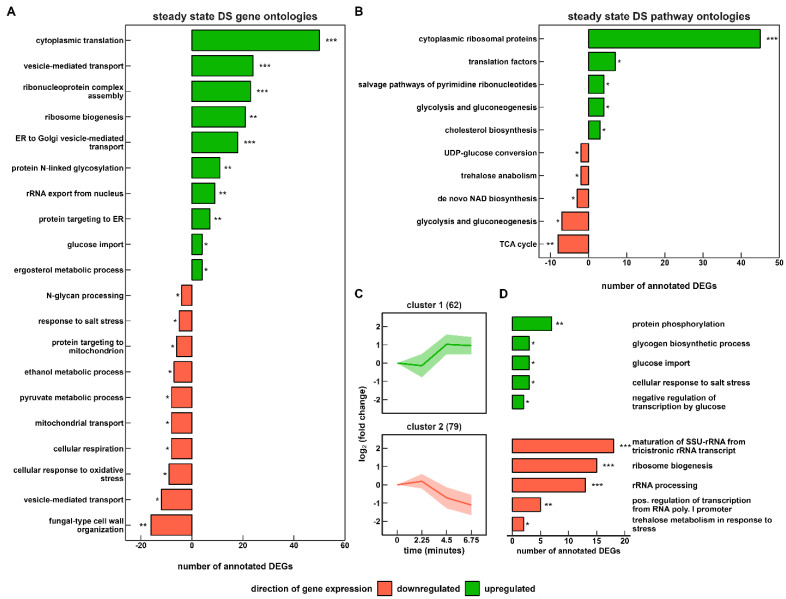
Functional enrichment analysis of DS based on (**A**) biological function and (**B**) pathway annotations. Short-term gene expression changes during the repeated (r) LSL cycles are shown in (**C**). Two k-means clustered groups of co-expressed genes are shown with the number of corresponding genes in brackets (**D**) representing the corresponding enrichment analysis of biological function. The false discovery rate (FDR) is indicated by asterisks for each category (* 1 × 10^−5^ ≤ FDR < 5 × 10^−2^; ** 1 × 10^−10^ ≤ FDR < 1 × 10^−5^; *** FDR < 1 × 10^−10^).

Next, we set out to investigate the adaptation status of ScER in the permanently dynamic environment. We assessed the steady-state gene expression profile of the DS versus RS. Thereof, we uncovered 332 induced and 265 repressed genes that were characterized using gene ontology and pathway enrichment (Figure 5A,B). In addition, 141 transcripts remained responsive, as they were repeatedly upregulated and downregulated within r-LSL cycles (Figure 5C,D).

Completely DS-adapted yeast cells revealed a strategy of increasing their internal translation capacities against recurring starvation signals. Significant overrepresentation of related gene ontologies such as “cytoplasmic translation” and “ribosome biogenesis” further indicated that this strategy occurred for two sub-groups: transcripts coding for ribosome subunits and their maintenance apparatus. Notably, 45 RPs of the total 57 RPs revealed permanent amplification. In contrast to the observations for the s-LSL cycle, r-LSL gene expression changes were backed by a 25% increase in the intracellular RNA content (Table 1). Pathway ontologies further indicated the marked upregulation of “translation factors” including the initiation factors eIF1, eIF2β, eIF4A, eIF4E, and eIF6. Notably eIF4A (also known as TIF2), which was induced 2.2-fold, plays a pivotal role in the early response of yeast towards acute glucose shortage by dissociating from the 48S pre-initiation complex [54]. Dissociated eIF4A caused the instant stalling of translation initiation, which appeared to be alleviated through its upregulation under the given conditions. Figure 5C,D further indicates the persistent short-term regulatory responses of gene products involved in ribosome biogenesis and more particular, rRNA processing in cluster 2. This functional group accounts for 25% of the steadily upregulated and downregulated portion of the transcriptome.

In addition to protein synthesis, we observed a highly significant differential expression of several genes annotated to protein modification and trafficking. The upregulated group “protein targeting to the endoplasmic reticulum (ER)” contains all four subunits of the signal peptidase complex (SPC), which is involved in cleaving signal peptides of secretory and membrane proteins during translocation into the ER [55]. Regarding protein processing activity in the ER, significant upregulation of *N*-linked glycosylation genes occurred, such as the asparagine-linked glycosylation (ALG) group (*alg5/6/8*) and the oligosaccharyltransferase (OST) complex (*ost2/4/5/6*). Induction of genes that are involved in the formation of GPI anchors was observed, too (see Supporting Information B, sheet 12).

Next, anterograde transport from the ER to the Golgi apparatus was stimulated through the induction of several coat protein complex II (COPII) elements, such as the GTPase Sar1 and the ER vesicle genes *erv29*, *erv41*, *erv14*, and *erv15*. COPII-coated vesicles transport membrane-bound proteins to the Golgi apparatus for the maturation of *N*- and *O*-linked glycosylation [56]. Despite the strong upregulation of 22 Golgi-transport genes, downregulated mannosyltransferase transcripts, represented by the ontology “*N*-glycan processing”, were the only significantly enriched DEGs with a Golgi-located protein modification function. At the end of the secretory pathway, we found upregulated sterol biosynthetic genes in both the gene ontology and pathway enrichment. This observation, however, seems rather counter-intuitive given that sterol synthesis is oxygen-dependent, rendering *S. cerevisiae* auxotrophic for this essential cell membrane component in anaerobic cultivations. In addition, a pronounced repression of “fungal-type cell wall organization” occurred, which included a set of genes such as the cell wall mannoproteins *cwp1*, *cwp2*, *tip1*, *tir3*, and *ccw12*—all functionally related to the downregulated mannosyltransferase capacities.

Yeast cells reportedly maintain branched tricarboxylic acid cycle (TCA) activity under anaerobic conditions to supply building blocks for growth [57], which was reflected by the small amounts of extracellular succinic acid (Table 1). Moreover, respiratory abilities are preserved in the absence of oxygen, too [58,59]. Here, we observed a significant downregulation of these auxiliary functions of respiratory pathways, such as mRNAs involved in the reductive (*MDH1*, *MDH2*, and *FUM1*) and oxidative (*ACO1*) TCA branches and repressed “cellular respiration”, “mitochondrial transport”, and “TCA cycle” ontologies. Regarding central carbon metabolism, repression also occurred on the level of pyruvate and ethanol metabolism. Gene-level investigation revealed that TCA influx mainly was hampered through pyruvate dehydrogenase (*PDA1*), pyruvate decarboxylase (*PDC6*), and aldehyde dehydrogenase (*ALD4*). This coincided with amplified fluxes towards fermentative pathways upstream of TCA. For instance, *ADH5*, which supports ethanol production [60], was induced 5.2-fold. Glucose uptake was also re-arranged to cope with decreasing extracellular availability through increased expression levels of the high-affinity transporter mRNA *HXT2/6/7* and the hexokinase 2. Non-glucose hexose transporters, such as mannitol and sorbitol scavenging Hxt13/17 were found to be dynamically expressed/repressed in cluster 1 during r-LSL transitioning.

Taken together, the adaption towards short-term limitation–starvation cycling encompassed the marked restructuring measures taken to aim at fostering growth. Translation-related genes were amplified at the expense of sacrificing reserve respiratory abilities and some non-specific stress response mechanisms, such as the “cellular response to oxidative stress” and the “response to salt stress”. As indicated in the gene expression profiles, the ATP-demanding formation of ribosomes was additionally supported by fostering ATP generation through ethanol fermentation.

### 3.5. Comparing Transcriptional Responses of s-LSL and r-LSL

**Figure 6:**
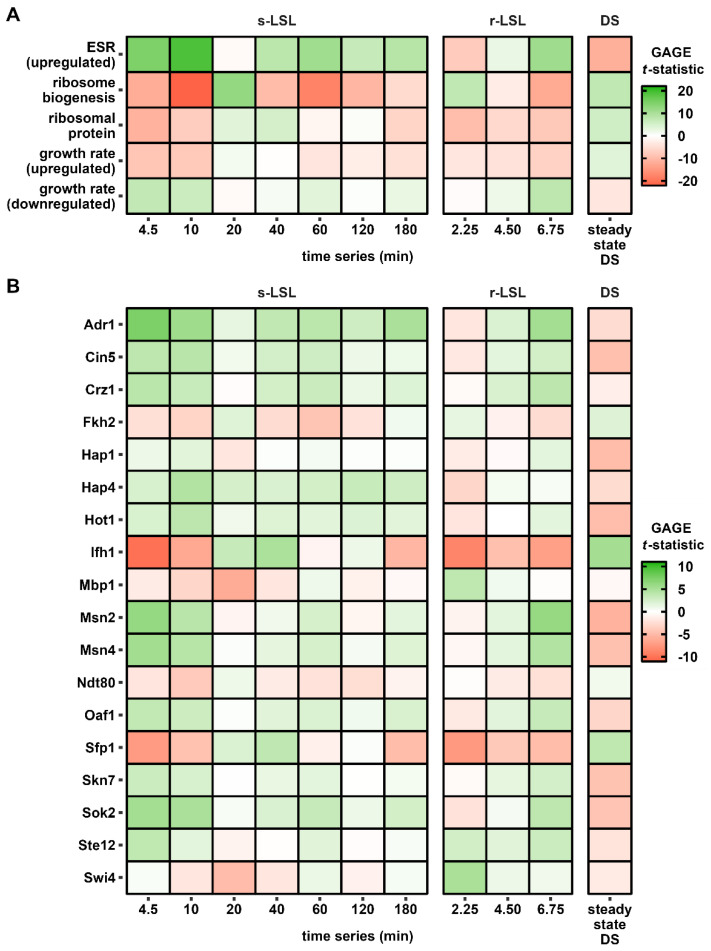
Gene set enrichment analysis (GSEA) of pre-defined gene lists from the literature (**A**) and transcription factor target lists (**B**). The reported *t*-statistic implies the strength and direction of the coordinated differential gene expression of a given set. GSEA was performed comparing the single (s) LSL time series against the reference steady state. Furthermore, the repeated (r) LSL time series was compared against its internal time point 0 min. The DS is a contrast between the reference steady state and all r-LSL sample points. Only gene sets with significant enrichment in at least one sample point (FDR < 1 × 10^−3^) are shown.

**Figure 7:**
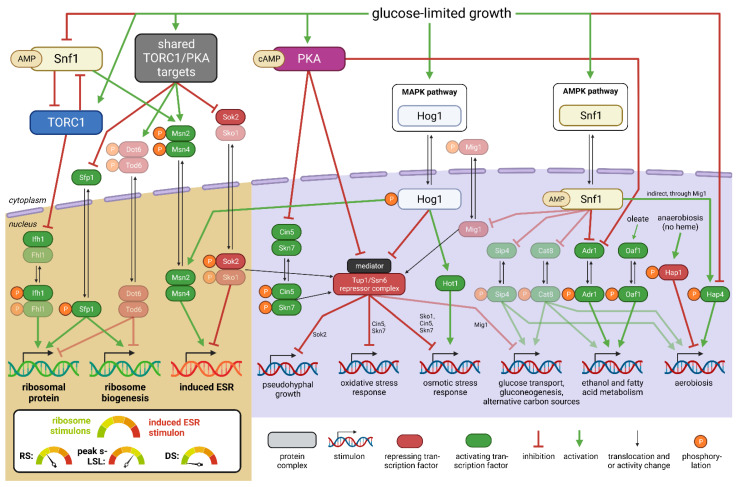
Regulatory kinase and transcription factor network active under the given experimental conditions. The brown background highlights the main ESR-associated stimulons. The violet background depicts observed co-induced stimulons. The simplified schematic network is limited to transcription factors (TFs) with significant calls in the GSEA and their known upstream effectors. Transparent TFs were either not significant or not part of the analytical pipeline but still included due to the reported implication in the given network. For simplification, not all pathway components, connections, and alternative functions are illustrated. The shown network is based on [63,64,65,66,67,68,69,70,71].

Regulatory information was deduced from gene set enrichment analysis (GSEA) to uncover the dynamics of literature-derived sets [61,62] (Figure 6A) and more nuanced transcription factor (TF)-mediated regulons (Figure 6B). The analysis was conducted on s-LSL, r-LSL and DS data while restricting the output to gene sets with statistically significant enrichment in at least one sample point across all conditions. Figure 7 serves as a visual summary of the involved regulatory elements and reported interactions.

ScER perceived the transient first-time exposure to starvation as an elicitor of the environmental stress response (ESR). As is typical for this program, the early induction of the ESR stimulon coincided with repression of ribosome stimulons represented by the RiBi and RP sets. Strictly growth-rate-dependent sets were considered to discriminate between the onset of the ESR program and mere adjustments of the growth rate. Since amplitudes measured after 10 min of s-LSL exposure of the ESR and RiBi sets showed 2–3-fold larger |*t*-statistic| values than those of the growth-rate-dependent sets, we concluded that the observed response was dominated by the ESR. This was also backed by constant *Y_DMB_*_/glucose_ throughout the non-adapted time-series. Similarly to the responses to the s-LSL cycle, r-LSL transcript dynamics reveal dampened oscillations, which are also visible in TF targets. Examples are the Msn2/Msn4 pair and the de-repression through Sok2 target genes. Further examples are also given regarding the repressed ESR branch with Ifh1-guided RP repression and Sfp1 control over both RP and RiBi genes [62,67].

Thus, a stress response–growth trade-off emerges, which is primarily balanced via the upstream effector target of rapamycin 1 (TORC1) and the protein kinase A (PKA). Our results further revealed additional signal input through the stress-activated signaling hubs, such as the mitogen activated kinase (MAPK) cascade or the glucose de-repression program [1]. One example is the osmo-responsive mitogen-activated Hog1 kinase as it is involved in both the direct induction of the osmotic stress response and fine-tuning of the ESR [67,72]. Hog1 activity was confirmed though the significant regulation of Hot1, a mediator of the osmo-specific gene expression program of this kinase. Closely related are the activators Cin5 and Skn7 and the repressor Sok2, which are stress-responsive recruiter molecules for the Tup1-Ssn6 repressor complex [73]. Tup1-Ssn6, in turn, interacts with the mediator complex—a coordinator of PKA and Hog1 signal integration under environmental stress [67]. Tup1-Ssn6 recruitment is not the only role of Skn7 as it further stabilizes the calcineurin-dependent transcription factor Crz1 [72]. Thus, it may not be surprising that both gene sets displayed almost identical expression dynamics. Another TF under MAPK control with a similar profile is Ste12, which regulates mainly mating and filamentous growth clusters [74]. Typical glucose de-repression signatures were observed by the deregulation of Snf1downstream targets, such as TFs Adr1, Hap4 and putatively Oaf1 [68,75]. Their activity can be observed in the transient induction of cluster 3 in Figure 4 with the enriched ontologies “cellular respiration” and “fatty acid catabolic process”.

GSEA further uncovered four active cell cycle-related transcription factors, two of which make up one partner of the heterodimeric SBF (Swi4) and MBF (Mbp1) factors that induce gene expression during the G_1_-to-S transition [76]. The forkhead homolog Fkh2 co-regulates genes which are active during both the mitotic and meiotic G_2_-to-M transition [77]. On the other hand, Ndt80 is a strictly meiotic regulator [78]. Apparently, cell cycle-related regulation followed a repressive pattern following the single stimulus. More precisely, the G_2_/M-related regulatory effects were found to be associated with the repressed dynamics of the ESR while regulation during G_1_/S took effect in a delayed manner, peaking after 20 min.

In conclusion, the transcriptional response to the s-LSL cycle comprised the transient expression of global regulatory programs, including the ESR and growth repression. DS-adapted yeasts showed the opposite gene expression pattern—namely, the repression of stress-induced gene sets and induction of growth-associated genes. Interestingly, there was still residual transcriptional activity of the same regulatory groups during the r-LSL time series. This result suggests that the short-term responsiveness of the regulatory circuitry controlling the adapted and non-adapted phenotypes was not entirely shut down during the DS.

### 3.6. Comparing Different Strain Backgrounds and Production Scenarios to the Same Stimulus

**Figure 8:**
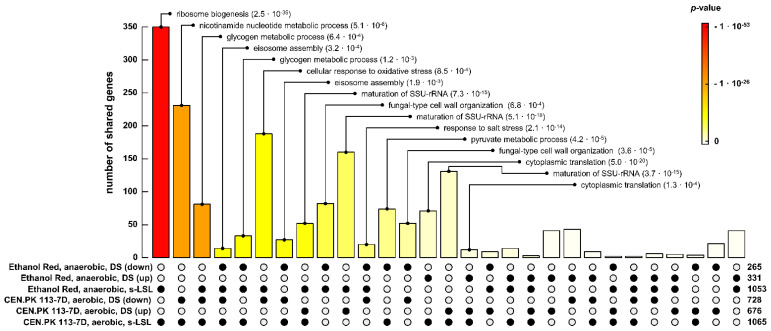
Shared sets of differentially expressed genes under anaerobic (this study) and aerobic [23] conditions in LSL-cycling chemostats. Black dots in the matrix indicate the considered DEG sets, the bar above represents the respective intersection size and the color gradient illustrates significance. The number of genes included in each set is reported on the right. Gene ontologies of the intersection sets are only reported if both the intersection and GO enrichment were significant (FDR < 1 × 10^−3^).

Transcriptional responses of ScER were compared to the likewise stimulated haploid MATa strain CEN.PK 113-7D [23] that was growing under aerobic conditions (Figure 8). Except for different |log_2_-fold change| thresholds, the same analytical pipeline was used in both studies.

In essence, commonly found key regulation features are (i) the trade-off between stress response and growth abilities and (ii) the re-allocation of intracellular carbon storage and RNA pools. Whereas CEN.PK 113-7D revealed the reduction of *Y_DMB_*_/glucose_ to a single LSL exposure, ScER merely limited responses to the transcriptional level. However, ScER significantly reduced its biomass yield after complete adaptation (r-LSL), which was not the case for CEN.PK 113-7D. Other differences were found for transcriptional relaxation times after single perturbation: ScER restored pre-perturbation conditions almost entirely within one hour. For comparison, the process lasted up to three hours within dampening amplitudes for CEN.PK 113-7D.

One-third of differentially expressed genes (350) of the non-adapted response was shared between both strains, indicating the presence of a highly conserved regulatory program. Shared genes in this core response were primarily enriched for RiBi mRNAs during the s-LSL cycle. Moreover, RiBi genes that were repressed initially upon unprecedented glucose exhaustion were upregulated later during the DS. This led to several overlaps of functionally related GO enrichments between the s-LSL cycle and upregulated DS sets of both strains. Eisosome assembly emerged as another conserved mechanism that is positively correlated to the ESR. The finding is in agreement with that of a recent study linking endocytosis to nutrient-scavenging activity in a nutrient-depleted environment [79]. As an analogy, “glycogen metabolic process” was found to be significantly enriched in overlapping gene sets of the non-adapted response and repression during DS. Stress programs with an unobvious role in surviving famine exposure, such as the oxidative and salt stress response, were observed to be downregulated during the DS in both strains, but only actively induced during the s-LSL cycle in ScER.

Peculiar CEN.PK 113-7D-specific gene expression changes occurred on the metabolic level as the ontology “nicotinamide nucleotide metabolic process” was mainly made up of glycolytic genes. Eighty-two genes were exclusively found in the s-LSL cycle and repressed DS datasets of ER and were functionally enriched for “fungal type cell wall organization”. Taken together, we interpret the overall sparsity of common gene sets across conditions per strain as further evidence of a highly conserved transcriptional regulation circuitry, which operates at the glucose limitation–starvation junction.

## 4. Discussion

### 4.1. Fluctuating Glucose Supply—Threat or Not?

Conditions of limiting glucose availability may frequently occur in large-scale fermentation processes [16,17,18,80] and are not restricted to aerobic cultivations [81,82,83]. In the present study, we cultivated ScER in a tightly controlled steady-state environment to investigate the effect of sudden glucose shortage. Since the stimulus was not strong enough to induce metabolic regime changes or to compete with maintenance demands, the observed signals may have mimicked the immediate cellular response to the environmental perturbations.

Apparently, the rapid ramp-down of the primary substrate supply instantly triggered the defensive transcriptional program, the so-called environmental stress response ESR [84], in the previously unstressed ScER strain. Thus, the first-time occurrence of glucose shortage was perceived as a ‘threat’. The primary task of the ESR is to save resources by ramping down growth capacities to invest in defensive precaution measures. Given the absent growth rate reduction and the relatively quick relaxation of the transcriptome changes, we reason that the ER efficiently shut down this program. Otherwise, said consequences would have been much more pronounced [1,23,85,86]. Comparing with CEN.PK 113-7D, trehalose mobilization, or the lack thereof, might contribute to this difference. The strain ScER instantly mobilized trehalose pools when the glucose influx decreased, likely to support energetic homeostasis [51]. Contrarily, CEN.PK 113-7D failed to maintain glycolytic flux as trehalose and glycogen pools remained stable during the stimulation (see Supporting Information A4). As a consequence, ATP reduction occurred in CEN.PK 113-7D, which further induced the ESR signaling cascade [23,87] through the energy-sensory Snf1 kinase. However, the role of trehalose in preventing energetic imbalances still remains somewhat elusive, though divergent short-term metabolic reactions to rapid changes in glucose concentration seem to cause strain-specific adaptation mechanisms. Glucose pulse experiments with *Escherichia coli*, *S. cerevisiae*, *Aspergillus niger* and *Penicillium chrysogenum* showcased this dependency [88].

Recurring glucose shortages did negatively affect the biomass-substrate yield. Even though biomass-specific ethanol production seemed to increase, the overall reduction in *Y_DMB_*_/glucose_ superimposed this effect and caused a net loss of the ethanol yield on glucose. Apparently, the regulatory modules involved during the peak ESR also controlled the DS phenotype since the same regulatory targets were affected in an inverse manner. Interestingly, these results showed high conformity with the operational mode of fast-growing yeasts, in which decreased carbon reserve pools enable increased anabolic demands to sustain enforced ribosomal machinery [45,89]. In an independent study, Metzl-Raz and colleagues confirmed that environmental sensing rather than internal feedback from the actual growth rate controls ribosome abundance [90]. Increased resource allocation for translation finally helps to accelerate growth after stress relief—a mechanism that displays an evolutionary advantage [91], especially in the selective environment of a chemostat.

Taken together, transient glucose depletion did not prove to be detrimental to the productivity of ScER. Nevertheless, we observed the presence of conserved regulation phenomena, such as increased transcriptional activity during ESR execution, which may cause unnecessary metabolic investments when stressful conditions arise [4,92]. Moreover, sustained transcriptional stimulation–repression dynamics after adaptation identifies a non-optimally operating biocatalytic host, and enables an understanding of the underlying regulatory mechanisms that motivated this study.

### 4.2. Not a Threat, but Still a New Habitat—How LSL Transitions Induce a New Growth Phenotype

The environmental stress response is a highly conserved gene expression program in *S. cerevisiae* [93,94]. Thus, it may not come as a surprise that different yeasts operating in different environments elicit strikingly conforming differential gene expression patterns when exposed to the same stimulus. The corpus of the induced ESR branch under acute stress, including glucose depletion, is driven by the feed-forward role of cAMP-dependent PKA signaling against the same targets primarily controlled by TORC1 under steady-state conditions [23,87,95,96,97]. When cAMP levels drop, PKA de-phosphorylates of the paralogue TFs Msn2 and Msn4 cause their nuclear translocation, during which they bind the so-called stress response elements within promoters to induce downstream expression [96]. In addition to the dual TORC1-PKA circuit, Msn2/4 regulation is fine-tuned in a condition-specific manner either upstream by the concerted activity of cross-talking kinases, such as PCK, Pho85, Hog1, and Snf1 or intersecting TFs, such as the repressors Sko1 and Sok2 [67,98].

Indeed, our transcriptomic analysis revealed the involvement of Hog1 through the significant activity of its osmo-specific transcription activator Hot1. This mitogen-activated kinase induces various mechanisms that converge for Msn2/4 regulation, including protection against temperature [99,100] and oxygen [101] shifts. The latter, especially, displays a high degree of coaction with the program induced by Hog1, even under anaerobic conditions [102,103]. Krantz et al. observed a dampened transcriptional response of osmotic and oxidative stress genes following a 0.5 M NaCl shock in anaerobic versus aerobic yeast cultures [102]. The authors inferred from their experiments that the glycerol production necessary to maintain NADH redox homeostasis in anaerobic cultures is the main driver of negative feedback for Hog1 phosphorylation. Conversely, this negative feedback does not exist in respiring, glucose-limited yeast cultures. Thus, the more stringent regulation of Hog1 during anaerobic growth might partially explain the more efficient ESR shutdown of non-adapted ScER compared to the dampened dynamic of CEN.PK113-7D during the s-LSL cycle. Consistent with this line of reasoning is the occurrence of overshooting Hog1 activity under aerobic conditions upon transient glucose withdrawal reported in an independent study [104].

The AMP-activated Snf1 cross-talk could not be characterized based on the dynamics of its actuating parameter, the adenylate energy charge, even though a rapid decrease was described under experimental conditions [105]. Upon glucose depletion, however, the Snf1 kinase acts as a cooperative modulator of the PKA pathway, with shared targets such as Msn2/4 and Adr1 [70], a direct regulator of glucose de-repression [70,106,107]. The role of Snf1 in the latter is thoroughly described in the literature to be involved during the diauxic shift when glucose is depleted in batch cultures [108,109,110]. During the s-LSL response, we observed a significant regulation of Adr1 and genes involved in ethanol metabolism and fatty acid degradation, a typical feature of the diauxic shift. Moreover, this transitionary phase usually encompasses the induction of respiratory, high-glucose-affinity, and alternative carbon assimilation activities [70]. Although we observed a differential expression of genes involved in said activities, our data lacked statistical significance for the strictly Snf1-dependent TFs Cat8, Sip4, and Mig1 [70,111]. A potential explanation might be the presence of Snf1 bypassing regulation through either PKA in the case of Adr1 or the heme-activated protein (Hap) complex. The Hap complex is also involved in glucose de-repression [112,113] but displays Snf1- and PKA-independent transcriptional regulation of respiratory genes [71,107].

Past studies concluded the same negative correlation between the induced ESR and repressed ribosome stimulons observed in this study during a s-LSL cycle [62,114]. Regarding the regulatory hierarchy, both stimulons are controlled by the overriding PKA activity under acute stress and both RiBi and RP genes are repressed through the de-phosphorylation of the master repressor pair Dot6/Tod6. A study by Lippman and Broach suggested that only Dot6 is a substrate of PKA under carbon source stress [107]. We were, however, not able to test for significant Dot6/Tod6 regulation as the target gene sets available from the Yeastract database were not rich enough to include the TFs in our analytical pipeline. Nonetheless, we identified significant regulation of the Dot6-antagonizing activators Sfp1 and Ifh1 [115]. The somewhat weakened response of the RP versus RiBi sets creates room for speculation as several potentially overlapping mechanisms might become relevant during the investigated perturbation. Both the Sfp1 and Ihf1 TFs target different promoter architectures. Nuclear exit causes Sfp1 release from RiBi-associated RRPE and PAC promoter elements while Ifh1 dissociates from an as-yet-unknown RP-specific promoter [115,116]. Acute glucose withdrawal further post-transcriptionally inhibits translation initiation [117], partly causing mRNAs to aggregate in so-called processing bodies or stress granules. Growth-associated transcripts are withdrawn from translation or actively degraded once located in said agglomerates [118,119]. Bresson et al. provide additional evidence that RiBi and RP genes are specifically flagged for TRAMP-mediated mRNA degradation following glucose withdrawal, with both gene sets exhibiting different degradation dynamics [120]. High PKA activity reportedly inhibits p-body and stress granule formation [121] and more remarkably, the assembly of these structures is independent of TORC1 or Snf1 signaling [122].

The results thus far indicate that cAMP-dependent PKA signaling is at the core of adaption toward dynamic glucose availability. Clearly, specific stress-responsive stimulons and the ESR were repressed while ribosome-associated mRNAs were induced. This emerging “high-growth” phenotype in the DS showed striking resemblance with the conditions of other experimental scenarios, all having high PKA activity in common [87,96,107,121,123,124]. Our experimental design, however, involved the same dilution rate during the DS compared to the RS, implying merely transiently downshifting and upshifting signals for protein kinase A. This was indeed reflected by the dynamic fraction of the transcriptome during the r-LSL cycle, especially with respect to the RiBi genes, which possess high transcriptional turnover [125]. However, the global transcriptional pattern displayed an overall elevated differential expression of PKA targets during the DS. Several feedback mechanisms may play a role when the PKA hub controls transcriptional responses. We recently speculated that during the LSL transition, disparate sensing of internal growth rate feedback and environmental substrate availability causes a boost of growth-related mRNAs at the end of the perturbation, also explaining the regulatory overswing that occurs during a s-LSL cycle [23,115]. When the population adapts to transitions in a recurring manner, a scenario might occur in which the molecular transmitter, cAMP, accumulates due to an asymmetry in production and decay [126,127], finally causing its levels to gradually ramp up during r-LSL adaptation. In conclusion, our data strongly suggest that PKA is the dominant factor that shapes the cellular fate in response to external glucose fluctuations in an industrial bioreactor setting in a highly conserved manner across different strain and bioprocess backgrounds.

### 4.3. Take-Away Message for Industrial Strain Engineers

We understand the obtained results to be fundamental proof that large-scale insight should be used in early-stage strain development. Here, even moderate environmental oscillations shifted the production host’s regulatory configuration. The resulting “large-scale-phenotype” might lead to non-optimal operational decisions given that most growth-coupled production processes are optimized based on the relationship between the biomass-specific production rate and the growth rate [128]. Obviously, an increased ribosome portfolio can impose a substantial metabolic burden on a cell, especially when the product is a heterologous protein [129]. In this specific context, strain performance seems rather unpredictable as neither prokaryotic nor eukaryotic hosts unanimously possess a linear correlation between ribosome content and protein productivity [130,131].

ScER was proven to be particularly robust under the investigated conditions. Nevertheless, its gene expression profile revealed the presence of futile stress-responsive mechanisms. The induced ESR branch during the s-LSL cycle and the repeatedly triggered RiBi cluster during the r-LSL cycle depict appealing targets for constructing a streamlined universal production chassis. For instance, Msn2/4 deletion could reduce induced ESR expression when cells adapt to emerging famine zones during fed-batch processes, whereas the antagonizing TFs Sko1 and Sok2 still repress the ESR in the adapted state. Likewise, we observed several cross-talking transcription factors activating non-specific stress responses such as the activation of the osmotic stress response or the activation of oxygen-dependent energy scavenging. Hot1 or Hap proteins could be targeted to reduce said expression programs and further enable safe transcriptional expenditures, even though the latter should be restricted to anaerobic production strains. More globally, the stabilization of Tup1/Ssn6-guided repression carries the potential to avoid premature glucose de-repression with possible metabolic impacts. Examples of the beneficial impacts of TF modulation on bioprocesses exist in the literature. For instance, Hap4 deletion increased fermentative capacity during cellobiose fermentation [132], and similar results were obtained through Cat8 deletion [133]. However, the cited studies reasoned that metabolic rerouting rather than gene expression savings caused the boosted productivity.

Here, we propose a valorization of the generated data centered around the idea that the ESR and other transcriptional stress response programs aggravate a variety of production scenarios, all induced by PKA activity (reviewed in [134,135]). In a bottom-up strategy, all TFs that are involved in futile or non-specific transcriptional stress responses such as Msn2/4, Hot1, or Hap4 should be individually tested for their potential to abolish said responses. Finally, such a procedure could lead to a stepwise optimization of the yeast’s regulatory landscape and ultimately reduce the maintenance demands made upon the introduction of short-term stimuli.

## Figures and Tables

**Table 1:** Process parameters comparing the reference steady state (RS) against the dynamic steady state (DS). RS parameter values are the means ± standard deviation of three biological replicates. The DS indicates averaged parameter values over one 9 min perturbation cycle of three biological replicates. *D*, dilution rate; *DMB*, dry matter of biomass; *Y*_i/j_, yield of compound i from j; *q*_i_, biomass specific rate of compound i; *c*_i_, concentration of compound i; n.a., not applicable; n.s., not significant (*p*-Value > 0.05).

Parameter	Dimension	Steady State RS	Steady State DS	% Change	Welch Test (*p*-Value)
*D*	h^−1^	0.098 ± 0.003	0.099 ± 0.003	+0.5	n.s.
*DMB*	g·l^−1^	5.06 ± 0.04	4.72 ± 0.17	−6.8	n.s.
*Y_DMB_*_/glucose_	mol_C_·mol_C_^−1^	0.114 ± 0.001	0.106 ± 0.003	−6.9	0.05
*q*_glucose_	mmol_C_·g*_DMB_*^−1^·h^−1^	32.1 ± 0.8	34.7 ± 1.2	+8.0	0.04
*q*_glucose,max_ ^1^	mmol_C_·g*_DMB_*^−1^·h^−1^	71.50	64.80	−9.4	n.a.
*K*_S_ ^1^	mmol_C_·l^−1^	6.19	5.47	−11.6	n.a.
*Y*_ethanol_ _/glucose_	mol_C_·mol_C_^−1^	0.488 ± 0.033	0.468 ± 0.026	−4.0	n.s.
*q*_ethanol_	mmol_C_·g*_DMB_*^−1^·h^−1^	16.7 ± 1.3	17.3 ± 1.4	+3.6	n.s.
*q*_carbon dioxide_	mmol_C_·g*_DMB_*^−1^·h^−1^	7.68 ± 0.25	8.47 ± 0.18	+10.3	0.01
*q*_glycerole_	mmol_C_·g*_DMB_*^−1^·h^−1^	2.70 ± 0.09	2.99 ± 0.12	+11	n.s.
*q*_acetic acid_	mmol_C_·g*_DMB_*^−1^·h^−1^	0.04 ± 0.00	0.04 ± 0.00	−2.1	0.03
*q*_succinic acid_	mmol_C_·g*_DMB_*^−1^·h^−1^	2.9 × 10^−2^ ± 4.7 × 10^−3^	3.6 × 10^−2^ ± 1.4 × 10^−3^	+25.7	n.s.
*q*_unknown carbon_	mmol_C_·g*_DMB_*^−1^·h^−1^	1.06 ± 0.53	1.81 ± 0.91	+71.2	n.s.
*c*_glycogen_	mmol_C_·g*_DMB_*^−1^	2.87 ± 0.16	1.65 ± 0.12	−42	1.0 × 10^−3^
*c*_trehalose_	mmol_C_·g*_DMB_*^−1^	1.84 ± 0.19	1.25 ± 0.52	−32	n.s.
*c*_RNA_	mg·g*_DMB_*^−1^	64.3 ± 2.5	80.7 ± 3.1	+25	2.0 × 10^−3^
C-recovery	mol_C_·mol_C_^−1^	0.98 ± 0.02	0.98 ± 0.02		

^1^ Estimated parameters (see Supporting Information A2 for details).

## Data Availability

The data that support the findings of this study are available from https://dataverse.nl/dataverse/minden-MDPIgenes.

## Supplementary Materials

The following supporting information can be downloaded at: https://www.mdpi.com/article/10.3390/genes14050997/s1, supporting information A; supporting information B. References [15,28,35,136,137] are listed in the Supplementary Material file.
