# Supplementary material for: Mimicked Mixing-Induced Heterogeneities of Industrial Bioreactors Stimulate Long-Lasting Adaption Programs in Ethanol-Producing Yeasts
Source: Genes (Basel). 2023 Apr 27;14(5):997. doi: 10.3390/genes14050997 (PMC10218636; doi:10.3390/genes14050997)
Supplement: Supplementary file 1 [file genes-14-00997-s001.zip › MDPI_genes_MINDEN_supporting_information_A.pdf]

SUPPLEMENTARY INFORMATION

Mimicked Mixing-Induced Heterogeneities of Industrial Bioreactors Stimulate Long-Lasting Adaption Programs in Ethanol-Producing Yeasts

Steven Minden[1], Maria Aniolek[1], Henk Noorman[2,3] and Ralf Takors[1,*]

[1] Institute of Biochemical Engineering, University of Stuttgart, 70569 Stuttgart, Germany
[2] Royal DSM, 2613 AX Delft, The Netherlands
[3] Department of Biotechnology, Delft University of Technology, 2628 CD Delft, The Netherlands
* Correspondence: takors@ibvt.uni-stuttgart.de

A1 ESTIMATION OF ETHANOL STRIPPING

Ethanol can be partially stripped in gassed fermentation processes leading to underestimated parameters such as qethanol or the carbon balance. We estimated a 4.8 % loss of ethanol in the liquid phase using the approach reported by [1]. First, the millimolar stripping rate from the system was calculated according to equation 1:

$$\dot{m}_{\mathrm{ethanol,G}} = \frac{q_{\mathrm{ethanol}} \cdot c_{DMB} \cdot \dot{Q}_{\mathrm{G}}}{K_{\mathrm{ethanol,L/G}} \cdot D} \tag{1}$$

, with:

$\dot{m}_{\mathrm{ethanol,G}}$ stripping rate of ethanol (3.26 mmol·h^{-1})

q_{ethanol} biomass specific ethanol production rate (7.98 mmol·g_{DMB}^{-1}·h^{-1})

c_{DMB} biomass concentration (5 g_{DMB}·L^{-1})

$\dot{Q}_{\mathrm{G}}$ volumetric gassing rate (25.5 L·h^{-1})

$K_{\mathrm{ethanol,L/G}}$ partition coefficient of ethanol between the gas and liquid phase (3125 L·L^{-1})

D dilution rate (0.1 h^{-1})

Then, the 4.8 % loss of ethanol in the liquid phase due to stripping was calculated by dividing the ethanol stripping rate by the ethanol production rate:

$$\mathrm{loss}\ (\%) = \left[\frac{\dot{m}_{\mathrm{ethanol,G}}}{q_{\mathrm{ethanol}} \cdot c_{DMB} \cdot V_L}\right] \cdot 100 \tag{2}$$

, with:

V_L liquid reactor volume (1.7 L)

A2 ESTIMATION OF $q_{\text{glucose, max}}$ AND K_{glucose}

As of to date, there are no published parameters for hyperbolic glucose uptake kinetics under anaerobic growth conditions available for Ethanol Red™. In order to characterize the stimulus, we conducted a parameter fitting of equation 3 to our experimental data. We repeated the reference steady state value between -600 and 0 seconds in 50 s intervals as a means to increase the weight of the steady state relative to dynamic data. For simulation and parameter fitting, the R packages *deSolve* v. 1.33 and *minpack.lm* v. 1.2.2 were used, respectively [2]. Glucose uptake was modeled according to:

$$\frac{dc_{glucose}}{dt} = \left(c_{glucose,feed} - c_{glucose}\right) \cdot D - \left(\frac{q_{glucose,max} \cdot c_{glucose}}{K_{glucose} + c_{glucose}} \cdot c_{DMB}\right) \quad (3)$$

, with:

$c_{glucose}$	glucose concentration (initial value: 5.2 $mmol_C \cdot L^{-1}$ in s-LSL and 5.6 $mmol_C \cdot L^{-1}$ in r-LSL)
$c_{glucose,feed}$	glucose concentration in feed reservoir (1366 $mmol_C \cdot L^{-1}$)
c_{DMB}	biomass concentration (5.06 $g_{DMB} \cdot L^{-1}$ in s-LSL and 4.72 $g_{DMB} \cdot L^{-1}$ in r-LSL)
$q_{glucose,max}$	maximum biomass specific glucose uptake rate (initial guess: 65 $mmol_C \cdot g_{DMB}^{-1} \cdot h^{-1}$)
$K_{glucose}$	glucose half-saturation constant (initial guess: 6 $mmol_C \cdot L^{-1}$)
D	dilution rate (0.1 h^{-1})

The famine stimulus within each perturbation cycle was implemented using an ifelse-loop where the dilution rate was set to zero during the feed-off time. The ordinary differential equation (3) was solved with the *ode()* function of *deSolve* (method = "ode45") and the parameters $q_{glucose,max}$ and $K_{glucose}$ were estimated by curve fitting via a Levenberg-Marquardt routine implemented in *nls.lm()* of the *minpack.lm* package (output and statistics in table S1). The simulated glucose levels versus experimental levels are shown in figure S1 A+C. Furthermore, an estimation of uncertainty was conducted. A random variable within the

mean square error between model and experimental values was computed for each data point with *rnorm()* to achieve a new simulated dataset. The parameters were then fit to 1000 simulated datasets and a 95 % confidence ellipse was drawn around the resulting parameter tuples (figure S1 B+D).

Table S1. Parameter estimation and statistics output from *nls.lm()*.

parameter	data set	estimate	standard error	t-value	Pr > | t |	p-value
$q_{glucose,max}$ (mmol$_C$·g$_{DMB}^{-1}$·h^{-1})	s-LSL	71.50	0.02	$8.9 \cdot 10^4$	$< 2 \cdot 10^{-16}$	< 0.001
	r-LSL	64.80	0.00	$3.5 \cdot 10^5$	$< 2 \cdot 10^{-16}$	< 0.001
$K_{glucose}$ (mmol$_C$·l^{-1})	s-LSL	6.19	0.03	1.39	$< 2 \cdot 10^{-16}$	< 0.001
	r-LSL	5.47	0.00	106	$< 2 \cdot 10^{-16}$	< 0.001

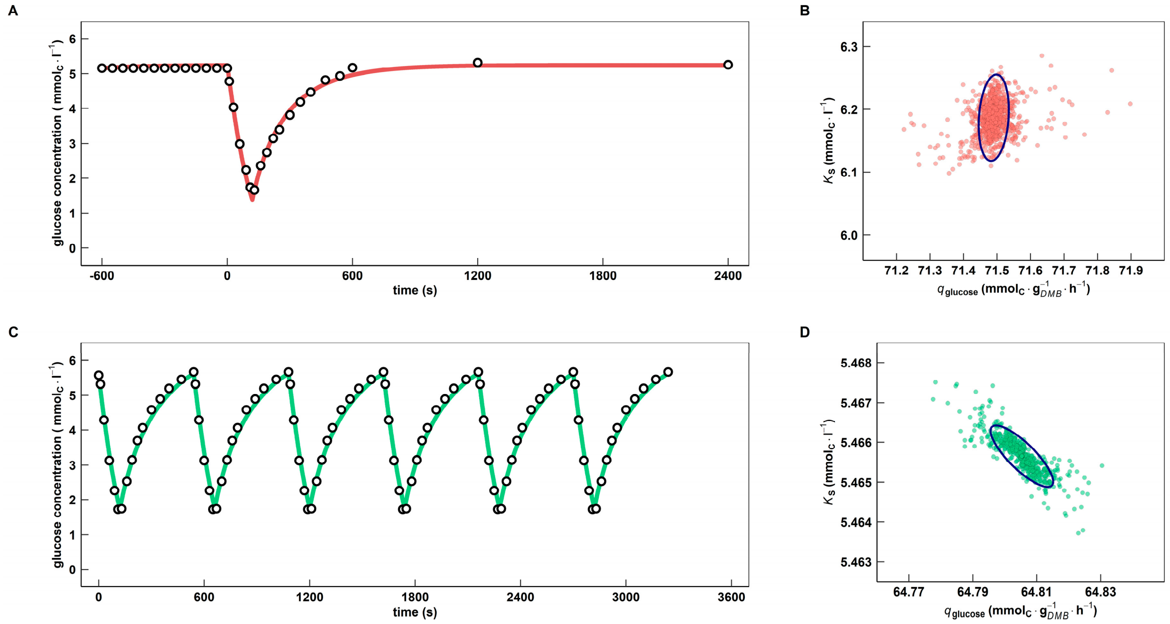

Figure S1. Simulation readouts for determination of glucose uptake kinetic parameters. Simulated data for s-LSL in shown in red (A) and r-LSL in green (C) with the corresponding experimental data as black circles. B and D depict the corresponding estimation of uncertainty. Estimated parameters for 1000 iterations over the curve fitting routine with simulated data, which was randomized within the mean square error of the model. The 95 % confidence interval is shown in blue.

A3 DIFFERENTIAL GENE EXPRESSION ANALYSIS

For gene expression analysis, the complete count table was split into three datasets: (i) the single-(s)-LSL time series including the reference steady state (RS) and post stimulus time points up to 3 h (figure S1), (ii) the characterization of the dynamic steady state (DS) using RS as the reference and all samples of the repeated-(r)-LSL time series representing DS (figure S2) and (iii) the r-LSL time series with timely equidistant samples within one perturbation cycle of 9 min with time point 0 s serving as the reference (figure S3). Except stated otherwise, all functions used in the subsequent section were called from the R package *DESeq2* v. 1.32.0 [3]. Cook's distances were computed for an initial outlier detection via calling "cooks" from the "DESeqDataSetFromMatrix" object of the respective dataset. In order to discriminate between the introduction of biological and technical variance, we included the variables time (for biological variance) and intervallic RIN (technical, for RNA integrity number) into the model. Next, count tables were transformed into the rlog space to stabilize the variance of genes with low counts using *rlog()*. Principal component analysis (*plotPCA*) revealed a strong influence of the RIN values (figures S2-S4 B+D). Thus, we applied a batch correction using *removeBatchEffect()* from the *limma* v. 3.48.3 package [4] to dampen the technically introduced variance leading to a reduced model, which allowed the investigation of biologically introduced variance (figures S2-S4 C+E).

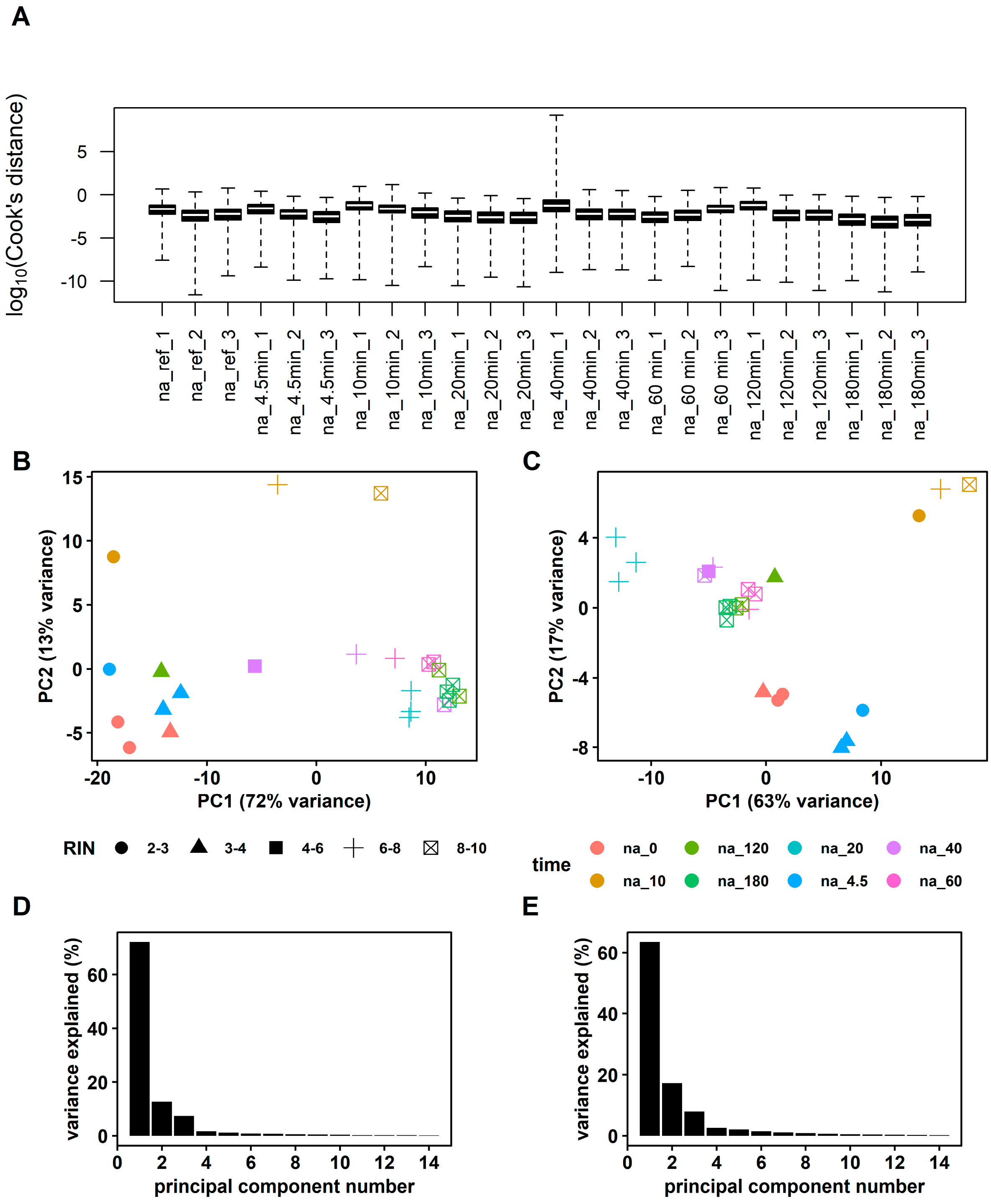

Figure S2. Analysis of all 24 samples from the s-LSL time series. (A) Boxplot of the Cook's distances. (B) Individual samples of 8 time points plotted on principal component 1 (PC1) and 2 (PC2). (C) Analogue to (B), but with the reduced model. (D) Corresponding scree plot of (B). (E) Corresponding scree plot of (C).

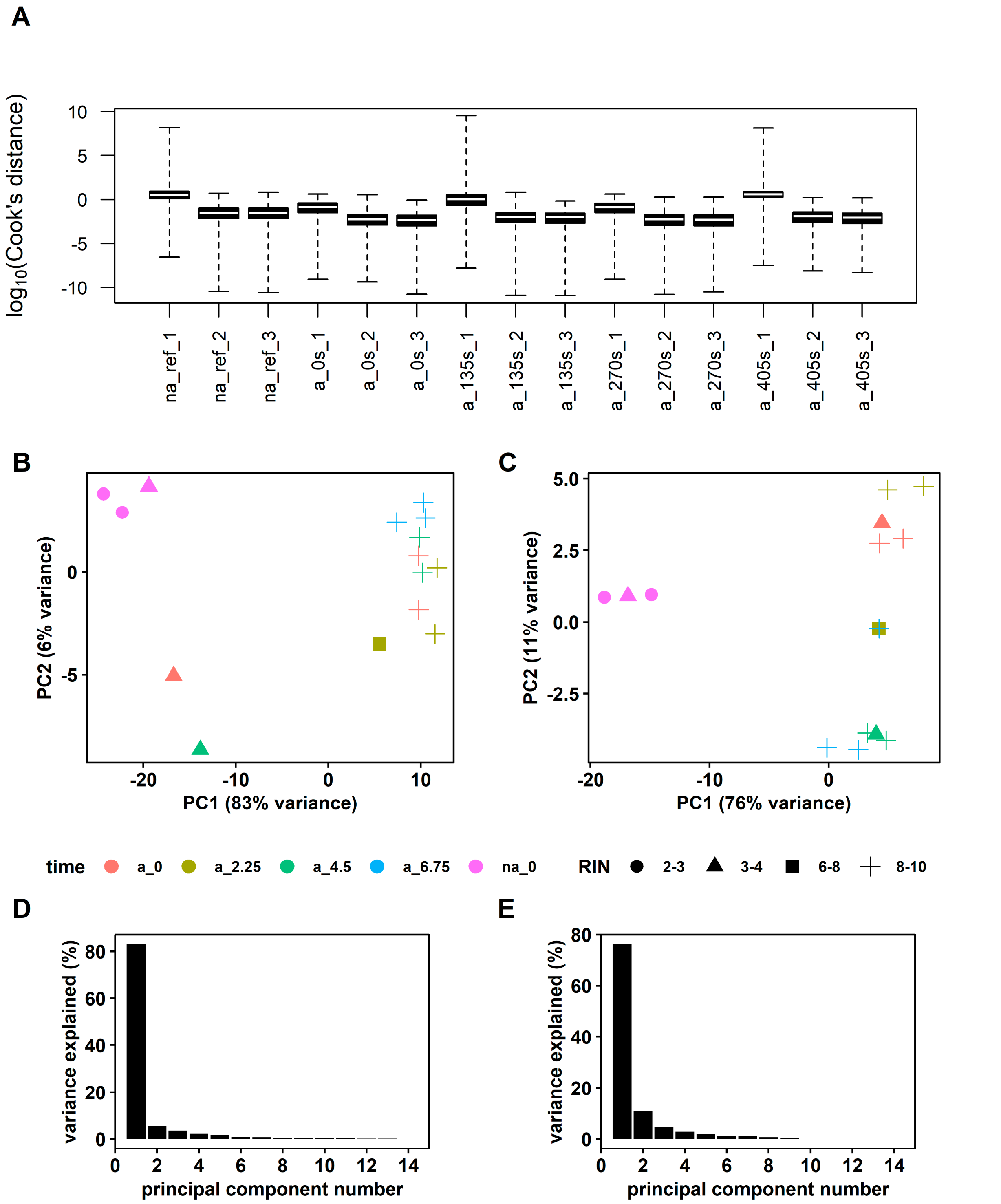

Figure S3 Analysis of 15 samples used for analyzing steady state DS (na_0 samples representing RS). (A) Boxplot of the Cook's distances. (B) Individual samples of 4 time points plotted on principal component 1 (PC1) and 2 (PC2). (C) Analogue to (B), but with the reduced model. (D) Corresponding scree plot of (B). (E) Corresponding scree plot of (C).

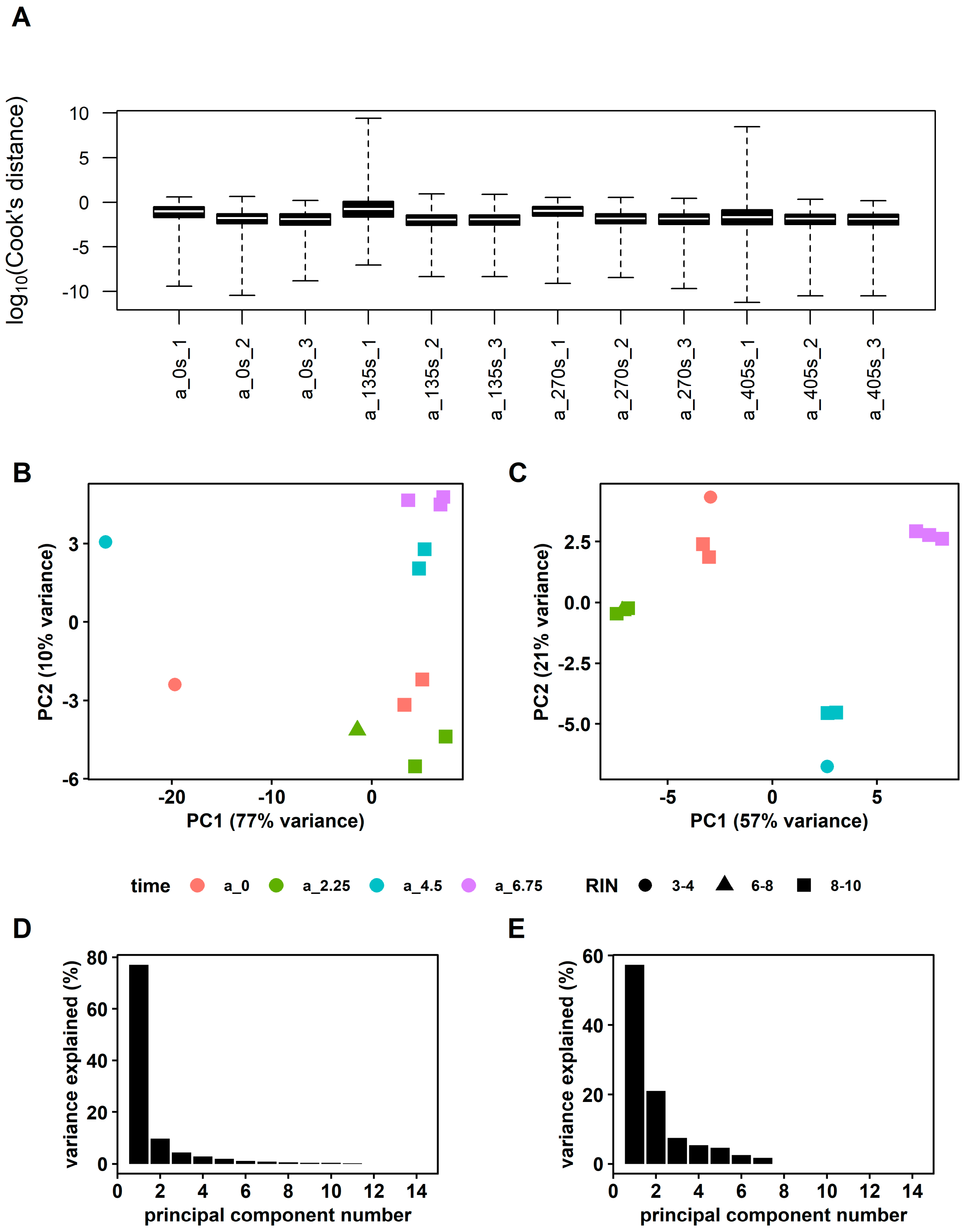

Figure S3. Analysis of all 12 samples from the r-LSL time series. (A) Boxplot of the Cook's distances. (B) Individual samples of 4 time points plotted on principal component 1 (PC1) and 2 (PC2). (C) Analogue to (B), but with the reduced model. (D) Corresponding scree plot of (B). (E) Corresponding scree plot of (C).

A4 COMPARISON OF STORAGE COMPOUND LIBERATION BETWEEN EXPERIMENTS

Differences of trehalose and glycogen dynamics between this and the aerobic experiment with CEN.PK 113-7D are visualized in the following figure S4. The comparing plots reproduce data from figure 3 for the 40-minute time window of the main publication and from reference [5] to ease comparing the underlying dynamics of the s-LSL response.

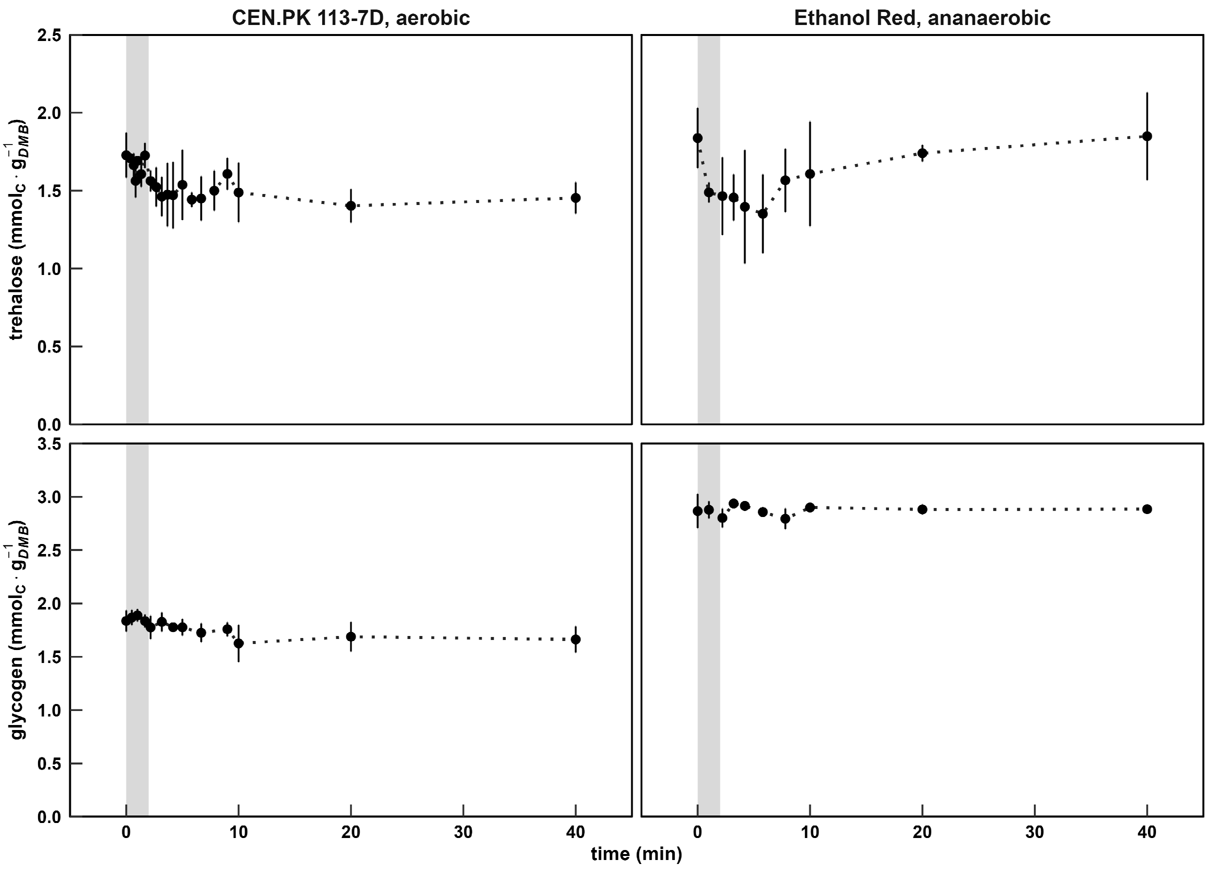

Figure S4. Characterization of intracellular trehalose and glycogen pools during the s-LSL stimulus. The left panel reproduces results from the aerobic stimulus-response experiment with CEN.PK 113-7D under otherwise identical experimental conditions [5]. The right panel reproduces results from the anaerobic experiment with Ethanol Red™ of figure 3 in this publication. The time series indicates dynamics following a single transition into starvation ("feed off" phase in grey). Time point 0 min is equal to the reference steady state. All values indicate means ± standard deviation of three biological replicates.

REFERENCES

1. Löser, C.; Schröder, A.; Deponte, S.; Bley, T. Balancing the Ethanol Formation in Continuous Bioreactors with Ethanol Stripping. *Eng. Life Sci.* **2005**, *5* (4), 325–332. https://doi.org/10.1002/elsc.200520084.

2. Soetaert, K.; Petzoldt, T.; Setzer, R. W. Solving Differential Equations in R: Package DeSolve. *J. Stat. Softw.* **2010**, *33* (9), 1–25. https://doi.org/10.18637/jss.v033.i09.

3. Love, M. I.; Huber, W.; Anders, S. Moderated Estimation of Fold Change and Dispersion for RNA-Seq Data with DESeq2. *Genome Biol.* **2014**, *15* (12), 1–21. https://doi.org/10.1186/s13059-014-0550-8.

4. Ritchie, M. E.; Phipson, B.; Wu, D.; Hu, Y.; Law, C. W.; Shi, W.; Smyth, G. K. Limma Powers Differential Expression Analyses for RNA-Sequencing and Microarray Studies. *Nucleic Acids Res.* **2015**, *43* (7), e47. https://doi.org/10.1093/nar/gkv007.

5. Minden, S.; Aniolek, M.; Sarkizi, C.; Hajian, S.; Teleki, A.; Zerrer, T.; Delvigne, F.; van Gulik, W.; Deshmukh, A.; Noorman, H.; Takors, R. Monitoring Intracellular Metabolite Dynamics in Saccharomyces Cerevisiae during Industrially Relevant Famine Stimuli. *Metabolites* **2022**, *12* (263), 1–26. https://doi.org/https://doi.org/10.3390/metabo12030263.
